# The contributions of value-based decision-making and attentional bias to alcohol-seeking following devaluation

**DOI:** 10.1111/add.12152

**Published:** 2013-04-04

**Authors:** Abigail K Rose, Kyle Brown, Matt Field, Lee Hogarth

**Affiliations:** 1 Department of Experimental Psychology, University of LiverpoolLiverpool, UK; 2 School of Psychology, University of New South WalesSydney, NSW, Australia

**Keywords:** Alcohol-seeking, attentional bias, concurrent choice, devaluation, social drinkers, value-based decision making

## Abstract

**Aims:**

To investigate the mediating role of attentional bias for alcohol cues on alcohol-seeking following devaluation of alcohol.

**Design:**

Between subject.

**Setting:**

Eye-tracking laboratory at the University of Liverpool.

**Participants:**

Student social drinkers (*n* = 64).

**Measurements:**

An operant choice task in which participants chose between simultaneously presented alcohol and non-alcohol drink rewards, while attentional bias for alcohol and non-alcohol drink cues was inferred from eye movements. Participants then consumed 30 mL of an alcoholic beverage, which was either presented alone (no devaluation: *n* = 32) or had been adulterated to taste unpleasant (devaluation: *n* = 32). Choice and attentional bias for the alcohol and non-alcohol drink pictures were then measured again.

**Findings:**

Alcohol devaluation reduced behavioural choice for alcohol (*F* = 32.64, *P* < 0.001) and attentional bias for the alcohol pictures indexed by dwell time (*F* = 22.68, *P* < 0.001), initial fixation (*F* = 7.08, *P* = 0.01) and final fixation (*F* = 22.44, *P* < 0.001). Mediation analysis revealed that attentional bias partially mediated the effect of devaluation on alcohol choice; however, the proportion of the variance accounted for by attentional bias is low to moderate (∼30%).

**Conclusions:**

Among student social drinkers, attentional bias is only a partial mediator of alcohol choice following devaluation of alcohol. Value-based decision-making may be a more important determinant of drinking behaviour among student social drinkers than attentional bias.

## Introduction

Since the publication of Robinson and Berridge's 1 incentive-sensitization theory it has been argued that attentional bias to drug cues (which develops owing to greater salience of the substance's rewarding properties and the predictive nature of the cue) plays a causal role in drug-seeking 2–4. Within non-dependent drinkers, alcohol-related attentional biases are greater in heavier, compared with light, drinkers 5, can be strengthened by priming doses of alcohol 6 and can predict drinking in university students 7. Using ecological momentary assessment techniques, Marhe *et al*. 8 found that drug-Stroop bias scores peaked just before heroin addicts relapsed.

Although such research implies that attentional bias reflects drug motivation, they provide no evidence for attentional bias being causal in drug-seeking. Initially, research that manipulated attentional bias and then measured this effect on drug-seeking suggested that attentional retraining could modulate drug-seeking 9. However, subsequent reports either failed to replicate, or produced null or partial effects (e.g. 10). Moreover, removing the attentional bias for drug-paired stimuli does not always reduce the stimuli's ability to motivate drug-seeking 11,12. This dissociation between attention and behaviour has been confirmed in animal work; the orienting response to reward-paired stimuli can be abolished by brain lesions, but the cues still motivate reward-seeking 13,14. These data suggest no causal role for attentional bias in drug-seeking.

Recently, researchers interested in value-based decision-making have employed choice procedures to measure the relationship between selective attention for reward cues, and the propensity to select between those rewards 15,16. In these tasks, participants choose between two reward pictures (e.g. potato chips/chocolate), while attention to these pictures is recorded by eye-tracking. These studies conclude that attention to a particular reward augments the propensity to choose that reward by ∼10%, while some central value-based decision process accounts for the remaining variance in choice/preference.

The aim of the current experiment was to employ a choice method to examine whether attentional selection between alcoholic and non-alcoholic drinks mediates the choice between those two rewards, following alcohol taste devaluation. We hypothesised that alcohol devaluation, relative to no devaluation, would (i) decrease alcohol choice behaviour and (ii) decrease attentional bias toward alcohol cues. The key aim was to determine whether a change in alcohol-related attentional bias (produced by devaluation) would mediate the change in alcohol choice, thereby indicating whether attentional bias for drug cues plays a causal role in drug-seeking. Given previous estimates, that attention to a reward increases choice by ∼10% 15,16, we hypothesised that the mediating effect of an alcohol-related attentional bias on alcohol choice behaviour would be low-to-moderate.

## Method

### Participants

Sixty-five participants (40 female) were recruited from the University of Liverpool. Owing to missing eye-tracking data, one participant was removed from all analyses (*n* = 64, 40 female). Participants [age: 23.52 years (SD ± 6.49)] were required to consume alcohol on a weekly basis [weekly alcohol unit (1 unit = 8 g alcohol) consumption: 15.11 (SD ± 13.93)], with no desire to reduce consumption. Participants were in good general/psychiatric health, were not taking any medication affected by alcohol, and had no history of major psychiatric disorders or illicit drug abuse/dependence.

At the point of recruitment, participants were asked to list their three favourite alcoholic and non-alcoholic drinks. Those who identified lager (a pilsner beer) or wine as a preferred alcoholic drink were recruited to ensure participants had experience of consuming the type of drinks depicted in the experimental cues.

The study received approval from the University of Liverpool Research Ethics Committee.

### Questionnaires

*AUDIT* 17: identifies hazardous and harmful patterns of alcohol use.

*Time Line Follow Back* (TLFB) 18: assesses typical weekly alcohol consumption [unit consumption (1 unit = 8 g alcohol)] using a diary format (2 weeks of drinking behaviour recorded).

*Desire for drinks*: desire to consume the available beverages (alcoholic/non-alcoholic) was assessed with a single question ‘How much would you like to drink this now?’ accompanied by a 100-point visual analogue scale (not at all to very much). This was also used to check for devaluation effects; we expected devaluation to decrease alcohol desire.

### Concurrent choice task

Developed from previous research (e.g. [19]), this task indexed preference for alcoholic relative to non-alcoholic drinks. On each trial, participants were required to press a key to win ‘points’ for one of two rewards (preferred alcoholic/non-alcoholic drink). Rewards were represented by two pictorial cues presented simultaneously on the left and right of the screen. For example, if a beer picture appeared on the left and a cola picture on the right, participants would press the left key to win beer points and the right key to win cola points. Participants were informed that more points would grant them a higher volume of that reward at the end of the experiment. In reality participants did not consume any drinks, but were compensated £8 for their time.

Picture cues were tailored to each participant, using the highest-rated available drinks [alcoholic (lager/wine) and non-alcoholic (e.g. cola/lemonade)]. Each photograph was taken in a semi-naturalistic setting (bar-lab), from the same angle, under the same conditions. The concurrent choice task was programmed using Inquisit 3.0.5 software.

In each trial, a central fixation cross was presented for 500 ms before two picture stimuli (preferred alcohol and non-alcohol) appeared on the left and right of the screen. The eye-tracker recorded fixations from the onset of the stimuli and continued to record until a response. From stimulus onset, participants were free to press the left or right response key to make their reward/picture choice. Each choice had only a 50% chance of yielding a point for the chosen reward (to increase switching between rewards). Thus, on a random 50% of trials when the alcohol stimulus was selected the outcome text ‘You win an alcoholic drink point’ would appear, and on the remaining trials ‘You win nothing’ would appear. The same was true after selecting the non-alcohol stimulus. Outcome texts were presented for 2000 ms prior to a random inter-trial interval (750–1000 ms). Over 60 trials, the left/right location of the alcohol and non-alcohol stimulus was counterbalanced. Sixty trials were completed once at baseline and once following the devaluation manipulation (see below).

### Eye-tracker

Attention to the choice stimuli was measured with an Applied Science Laboratories (ASL)-6000 remote eye-tracker (sampling rate 120 Hz). Data processing utilised ASL results software (v1.01; Applied Science Laboratories, Bedford, MA, USA). Measures were direction of initial fixation, last fixation prior to response, and dwell time. For each attentional bias measure, the proportion of observations to the alcohol picture relative to the non-alcohol picture was calculated 20. Gaze direction was measured in degrees, once every 8.5 ms. Fixations were defined as eye movements stable to within 1° of visual angle for at least 100 ms. Two areas of interest (AOIs) corresponded to the position of the alcohol and non-alcoholic pictures.

*Initial fixations* (initial orienting) 20 met the following requirements: (i) gaze was fixed on the central cross before drink stimuli appeared, (ii) eye movement occurred ≥100 ms after the drink stimuli appeared and (iii) eye movements occurred towards the AOIs.

*Last fixations* (attention prior to choice) were deemed to be the last fixation directed to an AOI prior to a response. Research suggests an increased likelihood of choosing a reward if it is fixated on last 16.

*Dwell time* (attention maintenance) was the time (ms) spent continuously fixating towards an AOI over the course of a trial. Research suggests an increased likelihood of choosing the reward fixated on most 16.

### Devaluation/no devaluation treatment

Participants consumed 30 mL of Becks lager or Blossom Hill wine (according to preference) which was unadulterated in the no devaluation (control) condition or adulterated with 0.6 mL of bitrex (0.256% solution) in the devaluation condition. Bitrex is used commercially to create a bitter-tasting liquid. This volume of bitrex equates to 50 p.p.m—half the limit added to household products to prevent consumption and has previously established taste aversion in humans 21. Evidence suggests that increased motivation for alcohol only occurs after moderate doses (e.g. ∼0.6 g/kg) 22; therefore, the consumption of 30 mL of unadulterated alcohol was used as a control condition.

### Procedure

**Figure 1:**
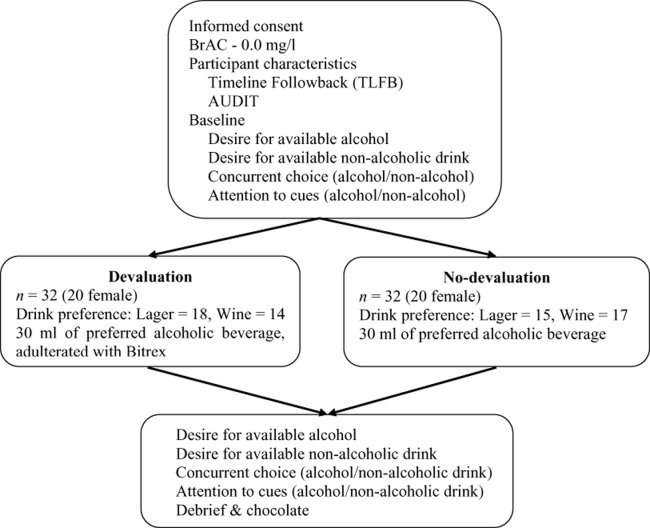
Procedure schematic. Participant characteristics were taken before baseline outcome measures were taken. Participants were randomised to the devaluation or no devaluation (control) condition (randomisation stratified by gender) before outcome measures were taken a second time

All testing took place between the hours of 12 pm and 6 pm. On arrival, participants were randomised to the devaluation or no devaluation condition [stratified by gender (condition *n* = 32)] and provided informed consent. A breathalyser reading of 0.0 mg/L was required. Participants completed the AUDIT and TLFB before providing baseline measures of desire to consume the available alcoholic/non-alcoholic beverages, concurrent choice and attention to the reward choice stimuli. Consumption of the adulterated (devaluation) or unadulterated (control) alcohol was then followed by a second drink rating and the choice task (with eye-tracking). Participants were debriefed and given some chocolate (to remove any bitter aftertaste) (see Fig. 1).

## Results

*t*-tests assessed whether participant characteristics differed across experimental condition. Analysis checked for any difference in drink preference (lager/wine) across conditions and whether preference affected the primary outcome measures. Effects of the devaluation manipulation (relative to control) on drink desire, choice behaviour and attentional bias were assessed using mixed design ANOVAs, findings were confirmed with follow-up *t*-tests. All variables met assumptions for parametric tests. Mediation analysis assessed whether the influence of devaluation on choice was mediated by attentional bias (using a composite score of first/last fixation and dwell time).

### Participant characteristics and drink preference

There were no differences between experimental conditions (*P*s > 0.1). Drink preference differed by gender, χ^2^ (1) = 24.73, *P* < 0.001; females were more likely to prefer wine (72.5%, *P* < 0.05) relative to lager, while men preferred lager (91.67%). Preference did not differ across condition, χ^2^ (1) = 0.56, *P* = 0.62 (see Table 1 and Fig. 1).

**Table 1:** Means (±SD) for participant characteristics by condition (*n* = 64)

	Mean scores			Statistics	
		
	No devaluation (control)	Devaluation	Overall	t	P
	20 female	20 female			
	12 male	12 male			
Variable					
Age	24.00 (7.46)	23.03 (5.41)	23.52 (6.49)	0.59	0.55
Audit	10.81 (5.29)	12.00 (6.46)	11.41 (5.89)	0.81	0.42
Weekly alcohol unit consumption (TLFB)	12.87 (11.45)	17.36 (15.89)	15.11 (13.98)	1.30	0.20

TLFB = Time Line Follow Back.

### Desire for drinks

Table 2 shows subjective desire for the experimental drinks before and after devaluation/no devaluation. Mixed ANOVA found an interaction between condition (devaluation/no devaluation) and time (baseline/test) [*F*(1, 62) = 12.09, *P* < 0.001, ŋ_p_^2^ = 0.16]; desire for alcohol decreased after devaluation [*t*(31) = 7.58, *P* < 0.001], but did not change after no devaluation [*t*(31) = 0.27, *P* = 0.79]. There was no condition effect at baseline [*t*(31) = 0.72, *P* = 0.47], but a condition effect was found after consumption [*t*(31) = 4.44, *P* < 0.001], reflecting lower alcohol desire following devaluation. Therefore, the devaluation technique successfully reduced the value of the alcoholic beverage.

**Table 2:** Means (± SD) desire for the experimental drinks before and after the no devaluation/devaluation treatment (*n* = 64)

	No devaluation		Devaluation	
		
	Before	After	Before	After
Drink type				
Alcoholic drink	49.09 (28.35)	50.28 (29.94)	53.78 (23.40)	20.44 (23.49)
Non-alcoholic drink	63.72 (26.61)	65.34 (26.50)	69.94 (25.00)	88.00 (18.64)

Desire for the non-alcoholic drink showed a significant condition by time interaction [*F*(1, 62) = 10.23, *P* = 0.002, ŋ_p_^2^ = 0.14], increasing significantly following devaluation [*t*(31) = 4.97, *P* < 0.001], but showing no change after no devaluation [*t*(31) = 0.45, *P* = 0.67]. The condition effect was non-significant at baseline [*t*(31) = 0.96, *P* = 0.34], but significant at test; desire for the non-alcoholic drink was greatest in the devaluation condition [*t*(31) = 3.95, *P* < 0.001].

Drink preference (i.e. lager/wine) did not influence the effects of the devaluation manipulation on desire (see Supporting Information).

### Behavioural choice

**Figure 2:**
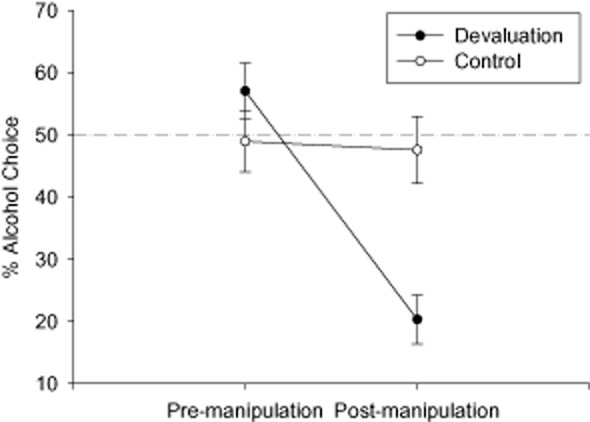
Proportion of responses for alcohol in the choice task over time, by condition (*n* = 64)

Figure 2 shows the percent of alcohol versus non-alcohol choice at baseline and test. A two-way interaction between condition and time [*F*(1, 62) = 32.64, *P* < 0.001, ŋ_p_^2^ = 0.35] demonstrated that alcohol choice decreased following devaluation [*t*(31) = 8.40, *P* < 0.001], but not no devaluation [*t*(31) = 0.32, *P* = 0.75]. The condition effect was non-significant at baseline [*t*(31) = 1.20, *P* = 0.23], but significant at test, reflecting reduced alcohol choice following devaluation [*t*(31) = 4.09, *P* < 0.001]. Thus, devaluation reduced choice of the alcohol stimulus. Drink preference did not influence this effect (see Supporting Information).

### Attentional bias

**Figure 3:**
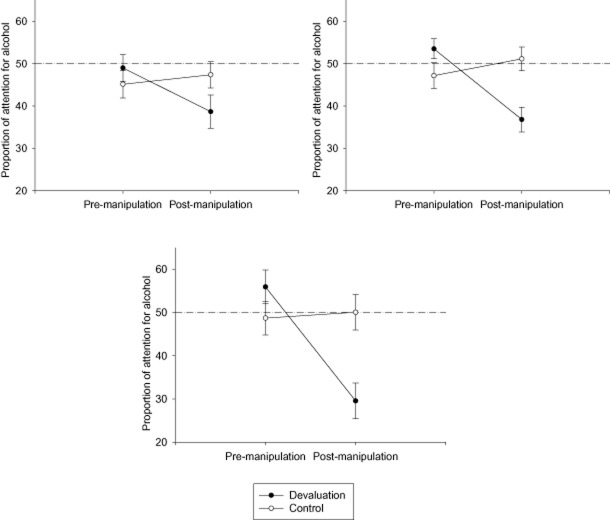
Proportion of first fixations (top left), overall dwell time (top right) and last fixations (bottom) towards alcohol images in the choice task over time, by condition (*n* = 64)

Figure 3 shows proportion of dwell time, first fixation and last fixation to the alcohol versus the non-alcohol stimulus at baseline and test of the choice task. Interactions occurred between time and condition for first fixation [*F*(1, 62) = 7.08, *P* = 0.01, ŋ_p_^2^ = 0.10], dwell time [*F*(1, 62) = 22.68, *P* < 0.001, ŋ_p_^2^ = 0.27] and last fixation [*F*(1, 62) = 22.44, *P* < 0.001, ŋ_p_^2^ = 0.26]. Compared with baseline, devaluation decreased the proportion of first fixations [*t*(31) = 3.10, *P* = 0.003, alcohol dwell time, *t*(31) = 5.44, *P* < 0.001] and last fixations [*t*(31) = 6.38, *P* < 0.001]. By contrast, there were no changes in the no devaluation condition regarding any attentional measure [*t*s(31) ≤ 1.29, *P* ≥ 0.20].

During baseline choice, there were no differences between condition for any eye-tracking measure [*t*s(31) ≤ 1.65, *P* ≥ 0.10]. During the test phase, differences between the devaluation and no devaluation conditions were marginal for first fixation [*t*(31) = 1.74, *P* = 0.09], and significant for dwell time [*t*(31) = 3.55, *P* < 0.001] and last fixation [*t*(31) = 3.51, *P* = 0.001]. Devaluation reduced attentional selection of the alcohol stimulus.

Drink preference did not influence the effects of the devaluation manipulation on attention (see Supporting Information).

### Mediation analysis

Mediation analyses assessed whether the change in the proportion of attention towards alcohol images (using a composite score of first/last fixation and dwell time) mediated the change in the proportion of alcohol choices. Change in attention and choice were operationalized as difference scores (post- minus pre-measures) such that increases in value represented an increase in the relative attention/choice for alcohol. As the causal steps approach to mediation has been criticised owing to its lack of statistical power and incorrect assumptions 23,24, an indirect effect approach to mediation was used 25. The approach calculates the indirect effect (through the mediator) and compares the relationship between the predictor and dependent variable before and after controlling for the indirect effects using a bootstrapping approach, which does not require a normal sampling distribution and minimises type 1 error through a reduced number of statistical tests 23,26.

**Figure 4:**
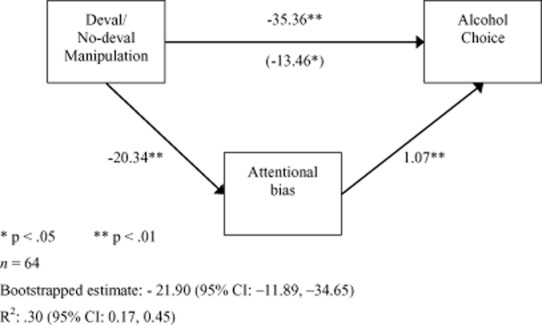
Path diagram representing how attention to alcohol images partially mediates the relationship between the drink value manipulation and choice behaviour. Values represent unstandardized coefficients for each pathway. The lower value on the top pathway (−13.46) represents the direct effect between the predictor and dependent variable after controlling for the whole indirect effect (which includes attention). Asterisks represent asymptotic estimates of significance. The R^2^ data indicates that the indirect effect accounts for ≤30% of the variance within the relationship between alcohol devaluation and alcohol choice

A representation of the model is shown in Fig. 4. Each value represents an unstandardised regression coefficient derived from a series of ordinary least squares regressions. It can be concluded that alcohol-related attention only partially mediates the effect of devaluation on alcohol choice, as the pathway between devaluation and choice remains significant after controlling for attention.

These conclusions are supported by the confidence intervals (of the indirect effect) provided from the bootstrapping analysis. The *R^2^* value highlights that the indirect effect of attention accounts for 30% of the variance in the effect of alcohol devaluation on subsequent choice. However, there is considerable debate regarding the usefulness of R^2^ data in establishing proportion of variance explained in mediation analysis 27,28. Given this, the R^2^ data must be interpreted with caution and, at most, we can say that the indirect effect accounts for ∼30% of the variance in the relationship between the devaluation effect and choice behaviour.

## Discussion

This study examined the change in alcohol choice and attentional bias for alcohol images following alcohol devaluation/no devaluation. As hypothesised, alcohol devaluation produced a reduction in alcohol choice, consistent with other data 29,30, while the no devaluation treatment produced little change in the experimental measures so can be accepted as a control. As hypothesised, devaluation also reduced attentional bias for alcohol images but, importantly, mediation analysis indicated that attentional bias plays only a partial mediating role in determining choice in social drinkers, and identifies this causal contribution as ∼30%. These results support our suggestion that central value-based decision processes may play a more substantial role.

The finding that attentional bias plays a small-to-moderate causal role in alcohol-seeking stands in contrast to studies that suggest that attentional biases are of no importance. Some human studies have found that cue-induced drug-seeking is not affected when attentional bias to the drug has been abolished 11,12. Similarly in animals, lesion-induced abolition of orienting to reward cues had no effect on the ability of those cues to motivate reward-seeking 13,14. This discrepancy might be because the previous studies 11–14 assessed the decision to respond (or not) following presentation of a single stimulus, which may not require attentional selection of the stimulus. In contrast, our participants chose between two matched rewards, which may rely to a larger extent on attentional selection between those rewards. This reliance is enhanced further in our design as participants were required to respond for a reward based on the reward's location, so some degree of attention was required to locate the desired reward and translate that knowledge to the correct response. This conclusion accords with the value-based decision theorists' view that attentional selection between two rewards has a reciprocal relationship with the emergence of a preference prior to choice 15,16.

A related issue concerns the estimate of the causal role played by attentional selection in choice, which value-based decision theorists estimate to be ∼10% 15,16. Given recent reviews on estimating proportion from R^2^ data, we can only say that the indirect effect accounts for ∼30% of the variance, and that our data provide the upper limit on the causal role of attention 27,28. Future research is needed to clarify this finding, identify other mediating factors and determine causal pathways more definitively.

Robinson and Berridge 1 originally suggested that the incentive motivation attributed to drug cues was crucial in driving drug-seeking. However, recent animal work has found marked individual differences in the degree to which predictive cues can trigger either sign-tracking (i.e. the animal approaches/interacts with the cue) or goal-tracking behaviour (i.e. the animal approaches the location in which the reward is delivered) 31. Both sign-trackers and goal-trackers administer the reward, suggesting that both value the reward. However, the cue may only develop incentive salience properties in sign-trackers. These findings suggest that the ability of predictive cues to develop incentive salience may differ across animals and that attentional bias to cues does not necessarily reflect the reward's value. Human research is needed to identify the individual differences that may support sign- or goal-tracking behaviour, as well as determining whether attentional bias to drug cues differ between these two behavioural types. Although we would suggest that our data highlight attentional selection as secondary to central value-based decision processes in mediating choice behaviour, more work is needed to identify which aspects of the cue and the reward determine choice. For example, future work may find that the mediating role of attentional bias is greater in sign-trackers compared with goal-trackers.

A further key issue concerns the value-based decision mechanisms underpinning alcohol choice in this paradigm, especially those underlying the reduction in choice following devaluation, which arguably play a causal role in drug-seeking. Learning theory provides several candidates that cannot be delineated directly with the current method Epub ahead of print. Reduced alcohol choice may have been goal-directed (determined by an evaluation of the current low value of alcohol) 33,34. Alternatively, the alcohol stimulus may have produced a weaker Pavlovian-to-instrumental transfer effect onto the alcohol response. However, this seems unlikely as such transfer effects are insensitive to devaluation 19,29. Finally, experience of the alcohol points may have produced an aversive state, modifying the propensity for the contextual cues to drive alcohol choice on future trials, through stimulus–response (S–R)/reinforcement or habit-learning. Devaluation effects readily modify choice under such conditions of contingent reinforcement 30. The most likely explanation is that alcohol devaluation reduced choice through a combination of goal-directed control and S–R/reinforcement learning 35.

It is important to note, that our sample (i.e. social drinkers) does not allow identification of the relative importance of attentional and value-based decision processes in dependent drinkers. The ambiguous results from initial investigations into attentional bias modification in dependent drinkers 36 indicate that future research needs to clarify the relative roles of attention and reward value in drinking behaviour in clinical samples. Our current sample had an average AUDIT score of 11, indicating hazardous drinking. This study offers translational evidence, and is an important step in the development of clinical studies and identification of future treatments which focus on manipulating drug value.

Although different theoretical perspectives (e.g. the low level of response and differentiator models) argue that individual differences in subjective alcohol effects influence drinking behaviour, the nature of this influence differs across models. Bartholow and colleagues 37 found greater attentional bias to alcohol cues in low-level alcohol responders, and argued that this bias illustrates motivation for the positive effects of alcohol, resulting in increased consumption. Alternatively, in support of the differentiator model, King *et al*. 38 found that in heavy social drinkers, sensitivity to alcohol's positive effects and greater measures of ‘liking’ and ‘wanting’ following an alcohol prime were associated with increased bingeing over 2 years. Our paradigm did not test these two models directly; however, we found a strong devaluation effect from aversive taste in social drinkers. The importance of taste, and, arguably, other hedonic attributes, may be greater in those hypersensitive, rather than hyposensitive, to alcohol effects, suggesting that different phenotypic risks factors are mediated by different underlying mechanisms. This is a cautious conclusion as our desire to drink measure only asked the participant how much they would like to consume the available drink—we cannot assume that all alcohol was devalued. Related to this issue is the fact that we did not include separate measures of ‘wanting’ and ‘liking’ of the rewards. Future research needs to identify the various elements of alcohol and the drinking experience that influence mechanisms underlying drinking behaviour (e.g. perceived value, decision-making, attentional bias), and determine whether such factors are more influential within certain populations.

In conclusion, value-based decision-making can drive alcohol-seeking in non-dependent drinkers and, while attentional bias mechanisms partially mediate this relationship, the magnitude of the causal role of attentional bias is likely to be low-to-moderate. The mechanisms by which value has its effect on choice, and the variables that affect value are important areas of research for understanding drinking behaviour.

## Supporting Information

**Appendix S1** Drink preference
