# Supplementary material for: The contributions of value-based decision-making and attentional bias to alcohol-seeking following devaluation
Source: Addiction. 2013 Apr 4;108(7):1241–9. doi: 10.1111/add.12152 (PMC3746131; doi:10.1111/add.12152)
Supplement: Supplementary file 1 [file add0108-1241-SD1.docx]

Rose AK, Brown K, Field M, Hogarth L. The contributions of value-based decision making and attentional bias to alcohol-seeking following devaluation

**Supplemental Information**

**Drink preference**

We assessed whether pre-existing drink preference may have affected the results. Chi square analysis showed that there was no significant difference between the number of participants choosing lager or wine as their preferred beverage according to condition, χ^2^ (1)= .56, p = .62 (see Figure 1). Although desire for drinks was significantly higher overall for those preferring lager (M = 49.16) relative to wine (M = 36.54), F (1, 60) = 4.98, p = .03, η_p_^2^ = .03, this did not interact with the devaluation manipulation over time, F (1, 60) = .11, p = .75, η_p_^2^ = .002.

There was no difference in overall choice for alcohol in those who preferred lager (M = 48.04) compared to wine (M = 38.94), F (1, 60) = 2.35, p = .13, η_p_^2^ = .04 and this did not influence the effect of the devaluation manipulation over time, F (1, 60) = .23, p = .63, η_p_^2^ = .004.

The proportion of initial fixation towards alcohol images for those who preferred lager (M = 48.20) was not significantly different from those who preferred wine (M = 41.38), F (1, 60) = 2.75, p = .10, η_p_^2^ = .04 and alcohol preference did not influence the effect of the devaluation manipulation on attention, F (1, 60) = .58, p = .45, η_p_^2^ = .01.

The proportion of final fixations towards alcohol images for those who preferred lager (M = 50.18) was not significantly different from those who preferred wine (M = 41.98), F (1, 60) = 2.89, p = .09, η_p_^2^ = .05 and alcohol preference did not influence the effect of the devaluation manipulation on attention, F (1, 60) = .20, p = .66, η_p_^2^ = .003.

The proportion of dwell time towards alcohol images for those who preferred lager (M = 50.14) was not significantly different from those who preferred wine (M = 44.15), F (1, 60) = 3.33, p = .07, η_p_^2^ = .05 and alcohol preference did not influence the effect of the devaluation manipulation on attention, F (1, 60) = .30, p = .59, η_p_^2^ = .005.
